# The Role of Antioxidants in the Interplay between Oxidative Stress and Senescence

**DOI:** 10.3390/antiox11071224

**Published:** 2022-06-22

**Authors:** Angelica Varesi, Salvatore Chirumbolo, Lucrezia Irene Maria Campagnoli, Elisa Pierella, Gaia Bavestrello Piccini, Adelaide Carrara, Giovanni Ricevuti, Catia Scassellati, Cristian Bonvicini, Alessia Pascale

**Affiliations:** 1 Department of Biology and Biotechnology, University of Pavia, 27100 Pavia, Italy; 2 Almo Collegio Borromeo, 27100 Pavia, Italy; 3 Department of Neurosciences, Biomedicine and Movement Sciences, University of Verona, 37129 Verona, Italy; salvatore.chirumbolo@univr.it; 4 Department of Drug Sciences, Section of Pharmacology, University of Pavia, 27100 Pavia, Italy; lucreziairenem.campagnoli01@universitadipavia.it; 5 School of Medicine, Faculty of Clinical and Biomedical Sciences, University of Central Lancashire, Preston PR1 2HE, UK; epierella1@uclan.ac.uk; 6 Emergency Medicine, Université Libre de Bruxelles, 1050 Brussels, Belgium; gaia.bavestrellopic01@universitadipavia.it; 7 Department of Internal Medicine and Therapeutics, University of Pavia, 27100 Pavia, Italy; adelaide.carrara01@universitadipavia.it; 8 Department of Drug Sciences, University of Pavia, 27100 Pavia, Italy; giovanni.ricevuti@unipv.it; 9 Biological Psychiatry Unit, IRCCS Istituto Centro San Giovanni di Dio Fatebenefratelli, 25123 Brescia, Italy; c.scassellati@fatebenefratelli.eu; 10 Molecular Markers Laboratory, IRCCS Istituto Centro San Giovanni di Dio Fatebenefratelli, 25123 Brescia, Italy; cbonvicini@fatebenefratelli.eu

**Keywords:** senescence, aging, antioxidants, oxidative stress, reactive oxygen species, minerals, flavonoids, vitamins

## Abstract

Cellular senescence is an irreversible state of cell cycle arrest occurring in response to stressful stimuli, such as telomere attrition, DNA damage, reactive oxygen species, and oncogenic proteins. Although beneficial and protective in several physiological processes, an excessive senescent cell burden has been involved in various pathological conditions including aging, tissue dysfunction and chronic diseases. Oxidative stress (OS) can drive senescence due to a loss of balance between pro-oxidant stimuli and antioxidant defences. Therefore, the identification and characterization of antioxidant compounds capable of preventing or counteracting the senescent phenotype is of major interest. However, despite the considerable number of studies, a comprehensive overview of the main antioxidant molecules capable of counteracting OS-induced senescence is still lacking. Here, besides a brief description of the molecular mechanisms implicated in OS-mediated aging, we review and discuss the role of enzymes, mitochondria-targeting compounds, vitamins, carotenoids, organosulfur compounds, nitrogen non-protein molecules, minerals, flavonoids, and non-flavonoids as antioxidant compounds with an anti-aging potential, therefore offering insights into innovative lifespan-extending approaches.

## 1. Introduction

It was 1961 when Hayflick and Moorhead introduced for the first time the concept of senescence [1]. Since then, a plethora of studies have been performed on this process, identifying highly complex and multi-step mechanisms leading to an irreversible cell cycle arrest, which can be initiated by various intrinsic and extrinsic stimuli, and developmental signals [2,3].

Distinct biological functions can be performed by senescent cells: from those beneficial falling under acute senescence to those dangerous falling under chronic senescence [4]. Concerning the beneficial functions, senescent cells guide tissue regeneration and embryonic development, limit tissue damage by reducing excessive cell proliferation and promote wound healing. Moreover, they encourage tumour suppression via upregulation of p53, p16 and p21 cell cycle inhibitors, or through production of interleukin-6 (IL-6) and IL-8. Finally, they play an important homeostatic role that is extremely dependent on their elimination by the immune system [5,6]. The senescence-associated secretory phenotype (SASP), the primary mediator of acute senescence, has the main role to signal the presence of senescent cells to the immune system and encourage their elimination. However, when senescent cells persist, their SASP profile becomes damaging, and this can transform senescent fibroblasts into pro-inflammatory cells, thereby promoting tumour progression [2,3]. 

Different molecular mechanisms are known to induce senescence [7]. Nuclear DNA damage is one crucial senescence mechanism, whose signals converge in p53 activation, which in turn induces cell cycle arrest. When the DNA damage response (DDR) is prolonged, it promotes senescence [4]. Further known mechanisms underlying senescence are: (1) persistent DDR activation at telomeres, the ends of chromosomes, which is sufficient to activate replicative cell senescence [8]; (2) oncogene activation partly via reactive oxygen species (ROS) production, determining hyperproliferation and altered DNA replication profiles [4,8]; (3) cell cycle arrest by upregulation of p21 and p16 [9]; (4) mitochondrial abnormalities with an increase in ROS synthesis and impairment in biogenesis and mitophagy [10]; (5) induction to resistance to apoptosis by upregulation of the antiapoptotic proteins [10]; (6) metabolic changes determined by senescence-associated-β-galactosidase (SA-β gal) accumulation along with the increase in cellular lysosomal content [10]; (7) large-scale chromatin reorganization occurring with the generation of senescence-associated heterochromatin foci, which suppress transcription of pro-proliferation genes [10]; (8) secretion of pro-inflammatory cytokines, chemokines, proteases, and growth factors that influence the neighbouring cells (SASP profile); (9) morphological alterations including cellular flattening and enlargement [10]; (10) post-transcriptional regulatory pathways taking place at different levels: through the action of mRNA-binding proteins (RBPs) and noncoding RNAs [11,12,13,14]; through a dysregulated splicing factor expression [12,15]; and through N6-methyladenosine (m6A) processes with specific m6A-binding proteins [14]. 

**Figure 1:**
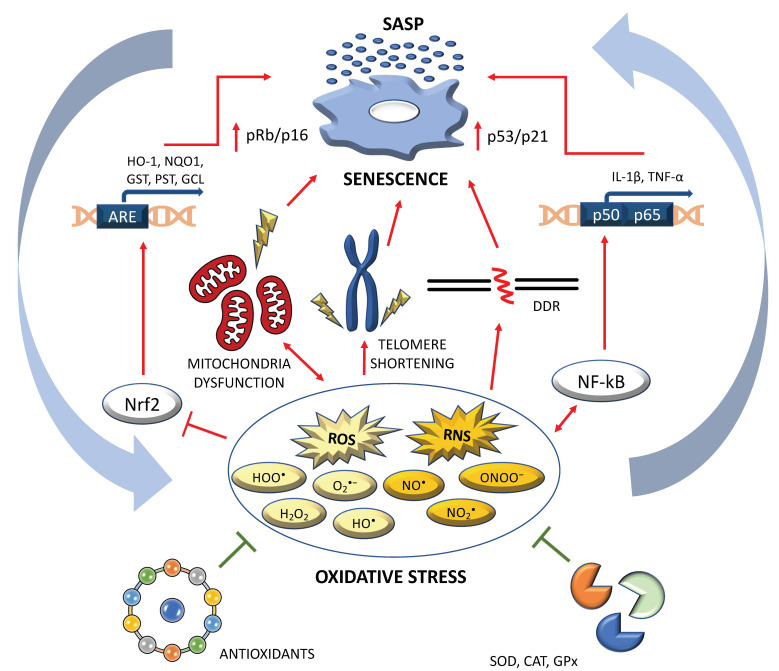
The interplay between oxidative stress (OS) and senescence. Excessive reactive oxygen species (ROS) and reactive nitrogen species (RNS) trigger senescence through different mechanisms: (i) via NF-kB stimulation, which induces the transcription of the main factors composing the senescence-associated secretory phenotype (SASP); (ii) through DNA double strand brakes, which trigger a sustained DDR response; (iii) via telomere shortening, which is directly linked to cellular senescence; (iv) through a double cross-talk between mitochondria dysfunction and ROS/RNS production and (v) via the inhibition of Nrf2, a crucial antioxidant transcription factor. Antioxidant molecules and antioxidant enzymes (i.e., superoxide dismutase, catalase and glutathione peroxidase) can counteract senescence through the inhibition of OS. Abbreviations: ARE: antioxidant responsive element; CAT: catalase; DDR: DNA damage response; GCL: glutamate cysteine ligase; GPx: glutathione peroxidase; GST: glutathione transferase; H_2_O_2_: hydrogen peroxide; HO-1: heme oxygenase-1; HO^•^: hydroxyl radical; HOO^•^: hydroperoxyl radical; IL-1β: interleukin 1β; NF-kB: nuclear factor kappa-light-chain-enhancer of activated B cells; NO^•^: nitric oxide radical; NO_2_^•^: nitrogen dioxide radical; NQO1: NAD(P)H quinone dehydrogenase 1; Nrf2: nuclear factor erythroid 2-related factor 2; O_2_^•−^: superoxide anion radical; ONOO^−^: peroxynitrite anion radical; PST: phenolsulfotransferase enzyme; SOD: superoxide dismutase; TNF-α: tumour necrosis factor α.

Increased oxidative stress (OS) is a further major driver of senescence [16,17,18,19,20,21]. The OS occurs when ROS/reactive nitrogen species (RNS) overproduction overwhelms the elimination ability of antioxidants. In a very recent exhaustive review [13], the authors summarized the major pathways inducing senescence through ROS/RNS deregulation. Specifically, a SASP profile can be promoted both by the failure of the antioxidant cascade due to defects in the well-known transcriptional factor Nrf2 (nuclear factor erythroid 2–related factor 2) [22], and by the activation of the redox-sensitive pathway influenced by another well-known transcriptional factor NF-κB [23]. A SASP profile can be further determined by the activation of molecular cascades linked to p53/p21 (due to persistent double strands breaks/telomere shortening), but also to p16/Rb (due to epigenetic modifications) [3,22,24,25,26]. Furthermore, an increase in ROS/RNS levels can be determined by mitochondrial dysfunctions, and this can contribute to telomere damage and epigenetic modifications [27]. Finally, alteration in the NAD^+^/sirtuin pathway can provoke senescence by the p53/p21 pathway, but it can also impact negatively on the specific functions of forkhead box O (FOXO) and peroxisome proliferator activated receptor γ coactivator 1α (PGC-1α), with consequent ROS increasing and mitochondrial dysfunctions (Figure 1). 

In this context, OS molecules could represent potential therapeutic targets to boost or delay cell senescence. Antioxidants compounds can be defined as senolytics, if they are able to selectively kill senescent cells, or as senomorphics, if they act by modulating the senescence phenotype [7,28]. Different mechanisms of action of senolytics have been reported in the literature: inhibition of the BCL-2 antiapoptotic family, negative modulation of the PI3K/Akt pathway and FOXO regulation [28]. On the other hand, senomorphics revert or slow down senescence by regulating the SASP [29]. 

Despite the considerable number of studies, a comprehensive overview of the main antioxidant molecules capable of counteracting OS-induced senescence is still lacking. 

**Figure 2:**
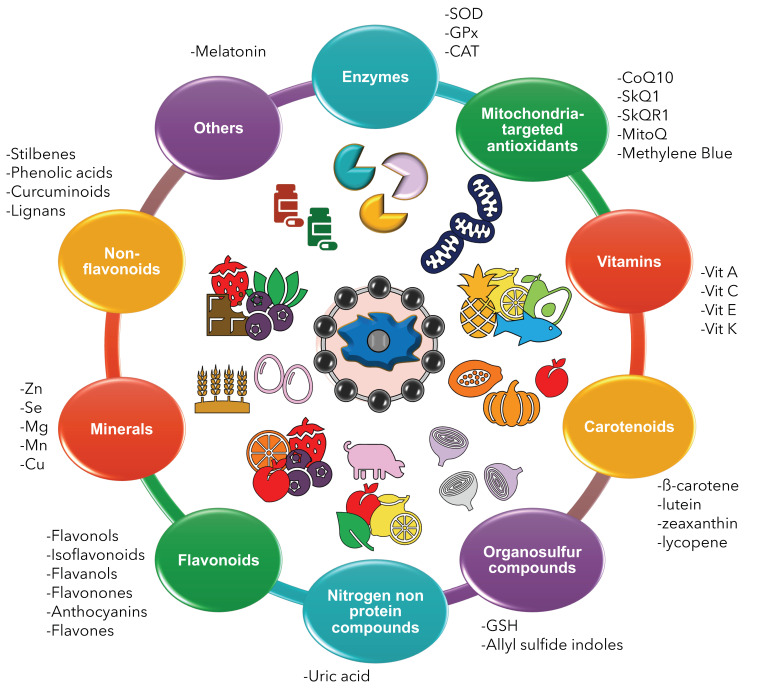
Antioxidants: classification. The figure illustrates the main classes of antioxidants capable of counteracting oxidative stress-induced senescence: enzymes, mitochondria-targeted antioxidants, vitamins, carotenoids, organosulfur compounds, nitrogen non protein compounds, flavonoids, minerals, non-flavonoids, and others.

In this work, we describe the role of enzymes, mitochondria-targeting compounds, vitamins, carotenoids, organosulfur compounds, nitrogen non-protein molecules, minerals, flavonoids, and phenolic acids as antioxidant compounds with an anti-aging potential (Figure 2). 

## 2. Results

### 2.1. Enzymatic Antioxidants

The term antioxidant refers to a wide class of molecules (bioactive substances and enzymatic complexes) that, present in small quantities (micronutrients) in the organism, can protect organic substrates, both natural (phospholipids, proteins, DNA) and synthetic (plastics, oils), from the attack of free radicals. All antioxidants inhibiting or reducing radical formation are acknowledged as preventive substances, as they work by preventing the formation of the so-called initiator radicals. In this group of molecules we can include: (a) chemical chelators, which are able, for example, to inhibit the Fenton reaction (Fe^2+^ + H_2_O_2_ → Fe^3+^ + OH^–^ + •OH); (b) sulphur and sulphide groups, which are able to decompose hydroperoxides in a non-radical way (i.e., ROOH + RSR → ROH + RSOR); (c) the antioxidant enzymes superoxide dismutase (SOD, EC 1.15.1.1) and catalase (CAT, EC 1.11.1.6), which break down superoxide anion and hydrogen peroxide, respectively.

Aging is a complex process where most antioxidant enzymes, including peroxidases, undergo a marked change [30]. The main endogenous antioxidants are enzymes that reduce the danger of free radicals, i.e., SOD, glutathione peroxidase (GPx) and CAT. In order to carry out their functions, these enzymes need trace elements such as selenium, copper, manganese and zinc and, for this reason, a daily intake of them is necessary. Raw foods, or nutritional supplementation, provide exogenous antioxidants such as ascorbate, tocopherol, vitamin C, β-carotenoids, bioflavonoids, lipoic acid, coenzyme Q10, selenium and zinc. These micronutrients should enable our cells to face ROS excess by promoting the antioxidant cellular endowment.

The superoxide dismutases (SODs) represent a wide group of antioxidant enzymes with complex activities [31,32]. Their activity has a dynamic nature, as they can change metal specificity to fit the different requests from cells in different microenvironments and functional conditions [32]. The role of SODs in aging has been recently addressed [33]. Particularly for skin aging, an event characterized by impaired wound healing, atrophy, reduced tensile strength and wrinkle formation, a marked loss in skin structural integrity and in collagen and elastic fibres, with weakening in the fibre network, has been reported, due to dysfunctional fibroblasts [34,35]. These senescent fibroblasts rapidly develop a growth arrest, changes in morphology and function, increased ROS production with a marked up-regulation of SOD2 in terms of both transcripts and proteomics [36,37,38]. The upregulation of SOD2 is induced in the senescent phenotype also in a paracrine way by physical insults such as UV irradiation [39] or by the immune release of chemokines, soluble factors, and cytokines from keratinocytes [40]. Upregulation of SODs might, therefore, mirror an impaired regulation of the cell survival machinery, to the point of even increase mortality in elderly patients [41]. The recent contribution by Mao et al. reported that, in 858 deaths investigated throughout a period of 6 years, a strong effect of sex (female) in the association between SOD activity and mortality was observed [41]. Furthermore, a decrease in SOD plasma concentration, particularly the isozyme SOD3, which is highly expressed in the arterial walls, can be detected along with further biomarkers of OS, such as AOPP (advanced oxidation protein products) and 8-iso prostane. Interestingly, the T-allele of rs2284659 in the promoter region of SOD3 has been related to a safer plasma redox balance, leading to an improvement in the cardiovascular outcome in patients with type 2 diabetes [42]. The same complex relationship between SODs and senescence, usually characterized by a SASP, namely an irreversible process of cell cycle involution alongside with a pro-inflammatory phenotype, deals with another complex actor of aging, the mitochondria biology [43]. It is well known that a deficiency in SOD2 in connective tissue leads to a senescent phenotype in bones, muscles and skin [44], whereas deletion in the gene expression of SOD1 leads to the appearance of a SASP marker in the kidney [45], yet many of these results should be associated with the biology of activated mitochondria. 

As cells and organisms increase their age, the respiratory chain tends to decrease, thus augmenting the release of electrons and reducing the generation of ATP. The theory of mitochondrial free radicals in senescence proposes that progressive mitochondrial dysfunction, which occurs with aging, results in an increased production of ROS, which in turn causes further mitochondrial and global cellular damage. This theory has been indeed reappraised in recent years [46]. 

When a switch from manganese (Mn^2+^) to iron (Fe^2+^) in SOD2 occurs, usually when due to a depletion Mn^2+^ is replaced by Fe^2+^, the new FeSOD2, which turns its function towards a pro-oxidant peroxidase, is a powerful causative factor of OS, mitochondria functional impairment and senescence [47]. A molecular cross-talk exists between Mn and Fe in mitochondria, able to switch SOD2 functionality [48,49]. This cross-talk may be impaired during aging, as, for instance, when fibroblasts accumulate iron during the development of a senescent phenotype [50], they may increase the Mn-Fe shift in SOD2, given that aging is also characterized by Mn and further micronutrients deficiency [51]. 

Catalase (CAT) is, most probably, a strong biomarker of senescence, due to the crucial role of H_2_O_2_ in modulating the OS response [52]. Further, peroxisomal OS is particularly crucial in the cell lifespan and survival ability and CAT plays an utmost role in this sense, so that CAT inactivation may lead, due to an impaired mitochondria-peroxisome cross-talk, to a condition of premature aging, also known as progeria phenotype [53]. Actually, hypocatalasemic fibroblasts show senescent-derived disorders [54,55]. 

### 2.2. Mitochondria-Targeted Antioxidants

The role of mitochondria in OS has long been established [56]. Due to the content of multiple electron carriers and an extensive antioxidant defence, they represent a key centre for ROS/antioxidant balance regulation [56]. Coenzyme Q_10_ (CoQ_10_), SkQ1 (a.k.a. visomitin), mitoquinone (MitoQ) and methylene blue are among the mitochondria targeted antioxidants exerting a role in counteracting OS-induced senescence, with CoQ_10_ being the most studied (Table 1 and Table 2) [57,58,59,60,61,62,63,64,65,66,67,68,69,70,71,72,73,74,75,76,77,78,79,80,81,82]. 

**CoQ_10_** is a lipid-soluble molecule involved in oxidative phosphorylation, metabolism, mitochondria permeability and antioxidant defence, either directly or indirectly [83]. A lack of CoQ_10_ has been related to several conditions, including aging and neurological disorders [83,84,85,86,87,88,89] (Table 1). A representative example is the increase in mitochondrial dysfunction, OS, apoptosis, and aging found in human dermal fibroblasts when CoQ_10_ production is pharmacologically inhibited [90]. Regarding senescence, CoQ_10_ deficiency has also been linked to increased p21 expression (a regulator of cell cycle progression), enhanced SASP production and downregulation of some extracellular matrix components (collagen type I and elastin) [66]. In vitro studies conducted on human skin fibroblasts exposed to H_2_O_2_ have shown that cell treatment with CoQ_10_ significantly reduced OS, decreased the amount of SA-β gal positive cells and restored collagen type I protein and the senescence-associated matrix metalloproteinase (MMP) production, therefore delaying skin aging [63]. Chronic treatment with nucleoside reverse transcriptase inhibitors (NRTI), which are clinically prescribed for the treatment of HIV, has been demonstrated to trigger oxidative damage, senescence, and endothelial toxicity. Recently, Chen et al. demonstrated that this phenotype could be reverted in vitro when human aortic endothelial cells are supplemented with CoQ_10_ [59], and similar findings were also reported concerning neural progenitor cells [65]. Stem cells are particularly sensitive to senescence induced by OS, as this condition may impact their self-renewal and repopulation capacity. In this respect, some in vitro studies indicate that CoQ_10_ can protect stem cells from OS-induced aging by influencing the Akt/mTOR signalling pathway, therefore preserving their proliferative balance [64]. 

In vivo administration of CoQ_10_ has long been known to improve immune functions by reducing immunological senescence that characterizes elderly mice [91]. More recently, studies conducted on mouse models of osteoporosis have demonstrated that CoQ_10_ supplementation (50 mg/kg/day) is sufficient to prevent osteoporosis by limiting ROS production and diminishing cellular senescence, both factors known to contribute to the disease development [62]. Moreover, CoQ_10_ may prevent cardiac aging, metabolic syndrome, and cardiovascular disorders when administered post-weaning to a rat model that mimics these conditions, and this improvement is mediated by the reduction of ROS and RNS, senescence, and apoptosis [58]. Similar beneficial effects have been obtained in cancer and Alzheimer’s disease (AD), in whose pathogenesis OS plays a predominant role [60,61]. Although beneficial, lifelong supplementation with CoQ_10_ may also be deleterious [92,93,94]. In this respect, results from a study designed to address CoQ_10_ administration only later in life showed that old mice subjected to a high CoQ_10_ diet displayed reduced OS in various tissues and were more efficient in performing the Morris water maze test compared to the untreated counterpart [95]. However, no improvements in other psychomotor or cognitive tests suggest that more research is needed to clarify the optimal timing of CoQ_10_ intake [95]. Nevertheless, the introduction of innovative delivery approaches to improve CoQ_10_ efficiency, such as the use of mitochondria-targeted nanoparticles, may represent a promising strategy to enhance CoQ_10_ antioxidant activity while limiting the possible side effects caused by high-doses administration [65]. 

CoQ_10_H_2_ (or ubiquinol), the reduced form of CoQ_10_, is even more efficient than CoQ_10_ itself in reverting senescence markers expression, both in vitro and in vivo [66,71]. The reason for this outperformance could be at least in part explained by a higher CoQ_10_H_2_ bioavailability at the same concentrations, therefore allowing a more efficient subcellular delivery [66,71]. For example, a study conducted by Huo et al. has shown that treatment with CoQ_10_H_2_ of H_2_O_2_-induced senescent human umbilical vein endothelial cells (HUVEC) is effective in reducing SA-β gal, SASP release and ROS production, but enhanced nitric oxide (NO) and endothelial NO synthase (eNOS) levels [67]. Diminished inflammation, OS-induced senescence and apoptosis have also been observed in the same cell line in other studies [69,72]. In vivo, experiments conducted on SAMP1 mice reported that ubiquinol administration at relatively high doses (250–300 mg/kg/day) for at least 10 months can reduce senescence grading scores and ROS production, while enhancing antioxidant defences [68,70]. However, no lifespan improvement was detected [68]. Upregulation of sirtuins 1 and 3 (SIRT1 and SIRT3), SOD2 and isocitrate dehydrogenase 2 (IDH2) enzymes, together with a higher reduced to oxidized glutathione (GSH/GSSG) ratio are also described upon dietary CoQ_10_H_2_ supplementation in an independent study, thus confirming the role of ubiquinol in protecting against cellular senescence progression and aging [73]. Finally, these improvements can be further enhanced by the combination of physical exercise and ubiquinol supplementation, as recently reported in in vivo studies carried out on SAMP8 mouse models [96]. 

**The SkQs** are a class of compounds made up by an antioxidant molecule (plastoquinone), a lipophilic cation and a linker moiety (decane or pentane). The family comprises SkQ1, SkQR1 and SkQ3, which belong to the mitochondria-targeted plastoquinone derivatives with antioxidant activity [97] (Table 2). In particular, SkQ1 and SkQR1 have been reported to reduce H_2_O_2_-induced senescence and apoptosis in vitro and to prevent senescence and tissue damage during aging [75]. Moreover, these benefits were achieved also in vivo in a wide range of age-related diseases and across species, even in the case of low doses administration later in life [74,76,97]. As for CoQ_10_, SkQ1 given to senescence prone rats at the concentration of 250 µmol/kg/day may be sufficient to prevent the physiological age-related deterioration of immunological defences [98]. Finally, AD-related cognitive decline, behavioural test scores and senescence-associated myocardial disease may improve in murine models upon long-term (lifelong) or limited (between 12 and 18 months of age) intake of SkQ1 [77,78,99,100,101]. Mechanistically, SkQ1 exerts its antioxidant properties by fatty acid co-mediated uncoupling, through interference with lipoperoxyl radicals and via regulating the electron flow at the level of mitochondria [102]. 

**Methylene blue (MB**) is a well-known mitochondria-targeted antioxidant that has shown promise in contrasting aging, especially skin aging [103] (Table 2). Methylene blue is reported to be particularly effective in delaying skin cellular senescence and extending fibroblasts lifespan in vitro, as well as in improving mitochondrial functions [79,80]. Although not yet fully understood, multiple mechanisms are thought to underlie its antioxidant function, including Keap/Nrf2 pathway upregulation, MB to MBH_2_ (the reduced form of MB) cycling in mitochondria, complex IV induction and increased production of collagen 2A1 and elastin, two components of the extracellular matrix fundamental for skin preservation [79,80,104]. Besides skin aging, MB may also prevent senescence and OS in other cell types, such as primary retinal ganglion cells and mesenchymal stem cells (MSCs), but its efficacy on stem cells remains limited to the cellular fraction characterized by a lower baseline level of OS [81,82].

Overall, despite promising evidence, results remain unclear. An important limitation is that studies are often performed on a specific cell line or murine model under certain conditions, which often prevent the results from being generalized and/or to be reproduced. In this respect, for example, data on extended fibroblast lifespan are debated, with some evidence showing that they can successfully decelerate aging and prevent senescence while other studies are inconclusive [105]. 

### 2.3. Vitamins

**Vitamin A**. Preformed vitamin A (all-trans-retinol and its esters) and pro-vitamin A (β-carotene) are essential dietary nutrients that provide a source of retinol, which regulate basic physiological processes [106,107]. Vitamin A and retinoic acid (a metabolite of all-trans-retinol) administration have been demonstrated to improve AD and age-related attenuation of memory/learning in mouse models, and this is probably due to their immunomodulatory effect and the reduction of pro-inflammatory cytokines and chemokines production by astrocytes and microglia, as well as to the promotion of differentiation of neural stem cells and regeneration of neural cells [108,109,110]. The role of vitamin A in the treatment of neurodegenerative diseases, such as amyotrophic lateral sclerosis (ALS) and schizophrenia, is currently under investigation [108,111]. Vitamin A has also been studied in association with quercetin, a well-known flavonol (see Section 2.7 Flavonoids) [112]. This combination has proven capable of reducing rapid senescence-like response induced by acute liver injury [113]. 

**Vitamin C** or ascorbic acid (AA) is a powerful antioxidant that can have beneficial effects on delaying the aging process and age-related diseases thorough its action on redox oxidative and mitochondrial pathways, on the immune system, on inflamm-aging, on endothelial integrity, and on lipoprotein metabolism [114,115,116,117,118,119,120,121]. Supplementation of AA also appears to prevent OS, immunosenescence, telomere attrition, disorganization of chromatin, and excessive secretion of inflammatory factors, and to prolong life [122]. For example, AA has been reported to extend replicative lifespan of human embryonic fibroblasts by restoring age-related decline of mitochondrial function and lowering cellular ROS, therefore reducing mitochondrial and DNA damages with decelerated telomere shortening [123,124]. Moreover, AA was found to have a protective effect also on human chondrocytes against OS by attenuating the increase of apoptosis, the loss of viability and the increase of senescence, and therefore hindering the development of osteoarthritis and aging of cartilage [125,126]. In the brain, AA has been increasingly found to promote several beneficial effects on neurodegeneration by direct neuroprotection against OS [116]. This vitamin has also been demonstrated to foster anti-senescence and anti-atherosclerotic effects via an improvement of lipoprotein parameters and microRNA expression through anti-oxidation and anti-glycation, especially in smokers [127,128,129]. Finally, a stable AA derivative, 2-O-α-glucopyranosyl-L-ascorbic acid (AA-2G), was also evaluated and compared with AA itself for its protective effect against cellular damage and senescence induced by hydrogen peroxide. The results suggest that the effect of AA-2G is longer-lasting compared to that of AA and this derivative might therefore be considered as a more stable form of vitamin C [130]. 

**Vitamin E** is a family of fat-soluble vitamins, which comprehends eight organic compounds with different degrees of antioxidant activity [131]. The impact of vitamin E on the prevention of chronic diseases is believed to be associated with OS and it has often been the subject of several studies. It has been recently observed that a higher consumption of antioxidants such as vitamin E is able to reduce ROS levels, leading to decreased telomere shortening, decelerating the cellular senescence, and potentially decreasing the risk of disease development [132,133]. 

**Vitamin K** compounds are a family of fat-soluble vitamins comprising structurally similar molecules including two main natural forms: phylloquinone (vitamin K1) and menaquinones (collectively known as vitamin K2). Besides being responsible for the activation of vitamin K-dependent proteins (VKDPs), which are involved in multiple functions such as bone and cardiovascular mineralization, vascular haemostasis, energy metabolism, immune response, brain metabolism, cellular growth, survival, and signalling [134,135,136,137], vitamin K appears to suppress the pro-inflammatory cytokines production through a non-carboxylative pathway, by modulating the gene expression of pro-inflammatory markers [138]. Accordingly, warfarin, a vitamin K antagonist, has been found to induce chronic low-grade inflammation in non-senescent vascular smooth muscle cells and enhance vascular aging and calcification, especially in young patients (<65 years old) [139,140]. 

### 2.4. Carotenoids

Carotenoids are naturally occurring lipophilic pigmented molecules found in fruits and vegetables with important antioxidant properties [141]. Chemically, their polyene structure, characterized by conjugated double carbon bonds, is at the basis of their ability to scavenge ROS and free radicals, therefore protecting from OS [141]. Although more than 750 carotenoids have been described [142], β-carotene, lycopene, lutein, and zeaxanthin remain the most examined for their implication in human health, with indications of their involvement in several age-related diseases [143,144,145,146,147,148,149,150]. There is evidence that carotenoids participate in the regulation of OS-induced senescence [151,152], and the same is true for parrodienes, which are structurally related to retinoids and carotenoids [153].

**β-carotene** is the precursor of retinoic acid [154,155]. Although it is generally considered an antioxidant, it can also function as a pro-oxidant compound depending on the circumstances, which are still not fully understood [156]. In vitro, keratinocytes treatment with β-carotene, prior to UVA exposure, prevents the upregulation of MMP-1, MMP-3 and MMP-10, therefore suggesting a protective role of β-carotene against OS-induced senescence [154]. 

**Lutein** and **zeaxanthin** are two macular pigment stereoisomers belonging to the xanthophyll group of dietary carotenoids [157]. Because of their unique ability to cross the blood-retina barrier, they accumulate in the macula and by virtue of their antioxidant, photoprotective and anti-inflammatory features are involved in the proper eye functioning [158,159,160]. A lack of lutein and zeaxanthin is generally associated to a poor cognitive performance in elderly [157,161]. Accordingly, improved cognitive functions were observed in elderly patients supplemented for one year with a mixture of lutein and zeaxanthin (12 mg/day), albeit not significant compared to the placebo group [162,163]. 

There is evidence that OS-induced senescence is involved in the pathogenesis of age-related macular degeneration (AMD), which represents the leading cause of blindness in aged individuals [158,164,165]. In this respect, Chae et al. documented that lutein treatment protects cells from H_2_O_2_-induced senescence by promoting the expression of antioxidant effectors such as nicotinamide adenine dinucleotide phosphate (NADPH) quinone dehydrogenase 1, heme oxygenase-1 (HO-1) and sirtuins (SIRT1 and SIRT3) [164]. Moreover, lutein and zeaxanthin intake, either as supplement or through xanthophyll-enriched foods, might delay AMD thanks to increased antioxidant protection [166]. Finally, data from Sen et al. show that a positive correlation exists between telomere length and xanthophyll carotenoids plasma levels, thus confirming the important role of lutein and zeaxanthin in the context of cellular senescence. 

**Lycopene** is a lipophilic carotenoid naturally found in tomatoes and other red vegetables and fruits with potent cytoprotective and antioxidant properties [167,168]. During aging, lycopene protects from cognitive impairment, insulin resistance and cancer, among the others [169,170,171]. In a study involving 1973 participants, Weber et al. showed that plasma lycopene levels are significantly different between young and old women, thus suggesting that its antioxidant activity is crucial to prevent age-related diseases [161]. Similarly, studies conducted on MSCs demonstrated that cellular pretreatment with lycopene protects against H_2_O_2_-induced senescence, enhances antioxidant defences (i.e., improved MnSOD activity and reduced ROS production) and prevents apoptosis through the modulation of Bax and Bak proteins [172]. When used alone, lycopene is known to foster the increase in HO-1, which is detected in dermal fibroblasts after exposure to UVA, thus representing a cytoprotective mechanism [173,174]. Moreover, the combination of lycopene with the anti-aging compound nicotinamide mononucleotide (NMN) has proven effective in reducing OS both in vitro and in vivo by enhancing the activity of SOD, CAT, GPx enzymes [175]. This effect, combined with the activation of the Kaep1-Nrf2 antioxidant pathway, efficiently prevents cells to become senescent, therefore confirming the promising role of lycopene in improving the anti-aging effect of already established compounds [175]. These results indicate that multiple carotenoids might be responsible for the antioxidant effects reported in the literature, but more research is needed to clarify the optimal combination of these supplements.

### 2.5. Organosulphur Compounds

**Glutathione** is a natural tripeptide, that is γ-l-glutamyl-l-cysteinylglycine, harbouring a fundamental role in the regulation of redox homeostasis [176]. Glutathione can exist in two forms: reduced glutathione (GSH) and oxidized glutathione (GSSG), which are converted into each other by the enzymatic activity of GPx (that links two GSH in one GSSG through the formation of a disulphide bond) and glutathione reductase (that catalyses the reduction of one GSSG into two GSH to the expenses of NADPH) [177]. Being the main intracellular antioxidant buffer, both the levels of GSH and GSH/GSSH ratio are tightly controlled through a fine regulation of their synthesis, metabolism, transport, and degradation [176,177]. A GSH deficiency has been related to the onset and progression of several diseases, including cancer, immunodeficiencies, seizures, neurodegeneration, cardiovascular dysfunctions, and diabetes [178,179,180]. As GSH levels can be used as a biomarker for the oxidative status of the cell [176,181,182,183], a reduction in GSH and the GSH/GSSG ratio are often reported during normal aging and in cellular senescence, both conditions influenced by OS [183,184,185,186]. For example, inhibiting or reducing GSH synthesis is sufficient to induce premature senescence and OS-mediated telomere shortening in HUVEC, and this condition is not restored by telomerase activity [187]. Similarly, a decreased activity of the enzyme glutamate-cysteine ligase, which is involved in the synthesis of GSH, has been linked to senescence, ROS production and DNA damage in primary mouse fibroblasts [188]. Of note, these detrimental effects are reversed by N-acetylcysteine supplementation, which is known to increase intracellular GSH levels [188]. Further evidence demonstrates that GSH deficiency can also trigger senescence through a pathway involving ROS production and Erk/p38 regulation, in a mechanism independent from the canonical p53 activation [189]. Therapeutically, small extracellular vesicles enriched in the glutathione S-transferase Mu 2 (GSTM2) enzyme, which works in conjunction with GSH to reduce OS and detoxify the cell from harmful compounds, can relieve senescence in various tissues when injected intraperitoneally in old mice [190]. Although reproducible, these results are not always consistent. Contrary to expectations, Tong et al. reported no reduction in brain GSH levels when analysing human postmortem brain samples in elderly subjects compared to younger ones, albeit the lack of data in living tissues represents an important limit of this study [191]. Moreover, Barilani et al. recently showed that increased GSH levels accompany MSCs aging [192]. Nevertheless, this mechanism might be a protective strategy to counterbalance the age-related increase in ROS observed during cellular senescence [191,192].

At brain level, elderly people (>74 years old) are generally characterized by reduced glutathione-S-transferase activity accompanied by slightly lower cerebrospinal fluid (CSF) antioxidant defences compared to younger individuals [193]. These data are consistent with previous evidence reporting an age-related decline in GSH levels both in the brain and the liver of SAM mice, along with other antioxidant molecules [194]. In humans, this impaired glutathione homeostasis might be involved in the pathogenesis of brain disorders, including age-related neurodegeneration [195].

Because GSH is a crucial regulator of oxidative status, it might also represent a promising therapeutic target. Indeed, enrichment analysis research performed on the DrugAge database, a repository of compounds known to extend life, showed that GSH is among the most common targets of lifespan prolonging drugs [196]. In this respect, Rebrin et al. demonstrated that the benefits of diets enriched in vitamins and micronutrients should be ascribed to increased plasma levels of GSH and improved mitochondria redox homeostasis in a sex and tissue dependent manner [197]. Direct GSH delivery is another therapeutic option. However, the insufficient bioavailability of GSH remains a limit, and the use of prodrugs and precursors of GSH have been proposed as an alternative route [198]. Recent data from Kumar et al. showed that supplementation with glycine and N-acetylcysteine ensures the correct GSH balance and extends mice lifespan by 24% [199]. Similarly, the administration of glutathione precursors (i.e., glycine and cysteine) is sufficient to significantly increase GSH levels and reduce OS in aged individuals [200,201]. 

Overall, these data point to GSH as a key antioxidant regulator involved in OS-induced senescence. However, although promising, more research is needed to carefully address its potential role as biomarker and therapeutic compound in the context of aging and senescence. 

**Alliin**, **allicin**, **allyl sulphides**, **allylcysteines** and other sulphur-containing compounds have long been known for their antioxidant properties [202]. Mainly contained in onion and garlic, they have shown to exert beneficial effects against cardiovascular diseases, cancer, aging, inflammation, OS, and infection, among the others [202,203]. Concerning aging, SAMP8 mice fed for 2 months with a diet containing 2% of aged garlic extract (AGE), which has been reported to have a higher antioxidant activity compared to fresh garlic extract [204], show improved lifespan and learning scores compared to the untreated counterpart [205,206,207]. The improvement in memory functions was then confirmed in vitro by a study conducted on primary hippocampal neurons derived from SAMP10 mice, whose dendrites are increased in length and number upon treatment with S-allylcysteine, the most abundant organosulphur compounds present in AGE [208]. In vivo, 12-week dietary supplementation with S-allylcysteine (0.05% or 0.2%) to 60-week-old wild type mice reduces senescence, improves mitochondrial functions, and ameliorates both aging and OS biomarkers [209]. At the molecular level, AGE reduces the production of ROS, increases glutathione levels, enhances the activity of the main antioxidant enzymes SOD, CAT and GPx, prevents lipid peroxidation and inhibits NF-kB (nuclear factor kappa-light-chain-enhancer of activated B cells) activity [203,210]. Despite encouraging results, discordant evidence emerged from some studies when the molecules contained in the AGE were tested individually. For example, while allicin shows senolytic activity when administered to breast cancer cells, alliin instead behaves as a pro-senolytic compound in the same conditions [211]. Still, when used as a whole, garlic extract exerts a strong NO scavenging function, reduces MMP-1 expression and ROS levels, inhibits SASP and improves SIRT1 activity, thus alleviating UVB-induced senescence in keratinocytes [212]. The combination of the beneficial effects exerted by the different AGE components may explain this discrepancy. For instance, recent evidence has shown that S-1-propenylcysteine, one of the AGE components, acts as an anti-inflammatory via stimulation of IL-10 expression and promotion of macrophage polarization towards an M2c status, which regulates the phagocytosis process of apoptotic cells [213]. According to these results, synergistic effects might be achieved by combining anti-inflammatory properties of S-1-propenylcysteine together with anti-aging and antioxidant activities reported for the other organosulfur compounds. Moreover, the administration dosage should be carefully evaluated because high concentrations of antioxidants may instead exacerbate OS. Overall, in line with the well-known beneficial effects of onion and garlic consumption, it is emerging that various organosulfur compounds commonly found in their extracts can prevent OS, thus supporting their usefulness in counteracting the aging process.

### 2.6. Nitrogen Non-Protein Compounds

**Uric acid** (UA) is a by-product of purine metabolism normally found in blood and urine. In the context of OS, although UA is classified as an important antioxidant molecule when circulating in the plasma, it exerts a potent pro-oxidant activity once inside the cell or in the form of extracellular crystals, probably due to different environmental interactions [214,215]. However, the molecular switch behind this dual role of UA, also defined as the “uric acid paradox”, remains largely unknown and controversial [216]. Accordingly, chronic serum hyperuricemia positively correlates with inflammation, DNA damage and OS, and has been implicated in the pathogenesis of several disorders, including renal, metabolic, and cardiovascular diseases [214,217,218]. Concerning senescence, several studies have demonstrated a link between UA levels, OS, and cell cycle arrest, both in vitro and in vivo, and improved aging-related functions have been observed following the administration of UA lowering agents [219]. For example, keratinocyte exposure to exogenous UA triggers cellular senescence and OS through a mechanism that is at the basis of the UV-induced damage [220], and a similar pattern has been reported for other cell lines [221]. Moreover, xanthine oxidoreductase, an enzyme involved in the production of UA, ROS and RNS, has been shown to promote aging and cellular senescence in vitro as well as in animal and clinical investigations [222]. Further, in vitro studies have reported an increased cellular senescence and enhanced ROS production in endothelial progenitor cells cultured in a medium containing UA at high concentrations (10 mg/dL) [223]. Of note, the same detrimental effects were shown in mice characterized by chronic hyperuricemia [223]. At the molecular level, there is evidence that UA triggers OS-induced senescence through the inhibition of the enzyme eNOS, which is essential to produce the scavenging molecule NO [215,223]. This condition triggers an OS imbalance, which promotes cellular senescence [215,223]. However, higher plasma UA levels in d-galactose rat models of accelerated aging were linked to decreased senescence and an increased SOD/(GPx + CAT) enzymatic ratio, which is indicative of antioxidant activity, thus confirming the beneficial role of UA when considered in the plasma [224]. 

In humans, results from a comparative study conducted on 26 elderly participants and 18 controls reported a 2-fold reduction in serum UA levels in aged individuals compared to controls, and this pattern was in line with diminished antioxidant defences [225].

Overall, these data show the existence of a correlation between UA and senescence. However, the dual role that UA may play in the context of OS should encourage further research to better clarify the befits and harms of UA-lowering agents. 

### 2.7. Flavonoids

Flavonoids are a class of polyphenolic secondary metabolites found in plants and are routinely consumed by humans. Chemically, they are polyphenols with the structure of a 15-carbon skeleton (C6–C3–C6) formed by two aromatic rings and one pyran ring [226]. Tea, wine, and Chinese herbal plants are the primary sources of flavonoids, as well as leaf vegetables, onion, apples, cherries, berries, soybeans, and citrus fruit [227]. Flavonoid compounds are divided into six subclasses, flavones, flavonols, flavanones, isoflavones, flavanols, and anthocyanins [228]. Beside the antioxidant activity, flavonoids have anti-inflammatory, vasodilator, anticoagulant, cardioprotective, anti-diabetic, neuroprotective, and anti-obesity activities, which make them of great interest as anti-aging compounds (Table 3) [228,229,230,231,232,233,234,235,236,237,238,239,240,241,242,243,244,245,246,247,248,249,250,251,252,253,254,255,256,257]. 

**Flavonols.** Quercetin is a flavonol known for its antioxidant, anti-inflammatory, antitumor, and senolytic properties [258]. Results obtained on different cell lines show that treatment with quercetin significantly lowers the levels of ROS and inflammatory cytokines, reduces the expression of SA-β gal, p16 and p53 and markedly increases that of the antioxidant enzymes SOD and CAT, regardless of the type of oxidative trigger used to induce senescence [258,259,260,261,262]. In addition to promoting the expression of Nrf2 [263], the beneficial action of quercetin appears to be mediated by the microRNA-155-5p, which is involved in the regulation of SIRT1 and NF-kB [262,264]. Moreover, as aging is associated with an inefficient protein-degradation (which is required to protect against OS), the effect of quercetin and its derivatives on the restoration of proteasomal functioning is of interest as rejuvenating strategy [265]. In trials in patients with diabetic kidney disease [266] and idiopathic lung diseases, quercetin induced a reduction in the expression of the aging markers p16 and SA-β gal, suggesting an anti-aging effect on kidney cells [267]. When combined with dasatinib (a tyrosine kinase inhibitor used as an antitumoral drug), quercetin showed senolytic activity, improvement of physical function and increased lifespan in mice [268]. Interestingly, as quercetin plus dasatinib treatment reduces intestinal senescence and inflammation while altering specific microbiota signatures, this optimized senolytic regimen might improve health via reducing intestinal senescence, inflammation, and microbial dysbiosis in older subjects [269].

Another promising bioactive flavonol with antioxidant properties is fisetin [270]. In vitro cell treatment with fisetin has shown a reduction in senescence, ROS, and apoptosis [270,271]. In vivo, 6-week oral administration of fisetin drastically reduced senescence, ROS, lipid peroxidation and protein oxidation in a rat model of induced aging, and lifespan extension has been reported in mice [235,272]. This positive outcome is due both to a senolytic activity of fisetin but also to its function as caloric restriction mimetic, which is reported to prolong lifespan [273,274]. However, the timing of fisetin administration seems to be crucial for obtaining a biological benefit. If on the one hand fisetin is protective when administered in the presence of OS, if it is given chronically in physiological conditions, it may even cause telomere shortening, therefore promoting senescence [275]. For this reason, more studies are needed to better assess the optimal conditions of fisetin intake and its mechanism of action. 

**Isoflavonoids.** Genistein is a phytoestrogen extracted by soya that is known for its antioxidant and anti-aging properties, although less potent than other flavonoids such as quercetin and kaempferol [264]. As for other antioxidants, the role of genistein is multiple: it can induce apoptosis acting as a cancer protective compound, but it can also reduce inflammation and OS acting as anti-aging and neurodegenerative protective agent [276]. Concerning senescence, genistein alleviates the genotoxicity and the cytotoxicity triggered by UVB exposure in human dermal fibroblasts [277]. Mechanistically, genistein reduces OS-induced senescence by mitigating the levels of mitochondrial ROS and of the DNA oxidation marker 8-OHdG, as well as by upregulating the SIRT1-FOXO3 axis, which is known to prevent aging [278]. 

**Flavanols**. There is consistency in the literature about the beneficial role of green tea on senescence-related mechanisms, thanks to its scavenging properties against ROS and RNS and its ability to stimulate autophagy [279,280,281,282]. These desirable effects derive from certain molecules known for their antioxidant role, mainly catechins [281,283]. Even if there are no conclusive results demonstrating the impact of green tea on the human diet, some studies investigated its effects on mice [284]. Catechins supplementation from green tea has been associated with a better memory performance and a protective role against DNA oxidative damage in SAM, independently from the age when the administration of green tea was started [285,286]. These antioxidants have a positive impact also on the brain structure, as murine models fed with green tea show an attenuated brain atrophy compared with SAM drinking pure water, thus suggesting an anti-aging property of these molecules [287]. (-)-Epigallocatechin-3-gallate (EGCG) is the most representative flavanol in green tea and its role in contrasting senescence is due to an activation of enzymatic and non-enzymatic antioxidative mechanisms (such as GPx and tocopherol), which are typically reduced in old age [288,289,290,291,292]. Interestingly, EGCG anti-senescence effects can also be observed at a macroscopic level as its supplementation reduces age-related sarcopenia in mice [293]. Nonetheless, an excessive amount of green tea has been associated with oxidative damage, underlying the need of further research to set the beneficial dose range [279,294].

**Flavanones** and **flavones**. Among flavanones, hesperidin is an antioxidant that can be typically found in citrus fruits [295]. Its properties impact positively on cardiomyocytes as it attenuates senescence-related oxidative damage, both independently and in combination with other molecules, through the induction of Nrf2 and of GST expression [247,296,297]. Citrus juice, which is rich in hesperidin and other flavanones as well as in flavones, anthocyanins, and other molecules, was reported to reduce ROS levels and reduce SA-β gal positive HUVEC [298]. Citrus fruit also contains another flavanone useful to counteract the effects of aging on myocardium, which is naringenin [299,300]. A recent study conducted in aging murine models suggests that the antioxidant properties of naringenin deriving from the activation of PI3K/Akt/Nrf2 pathway could greatly ameliorate both behavioural and neurological dysfunctions. The authors reported that naringenin administration markedly stimulated the activity of Nrf2 and improved the expression of the antioxidant enzymes HO-1 and NADPH-quinone oxidoreductase 1 [301]. 

Besides containing flavonones, citrus peels are also rich in flavones. One of these, nobiletin, was demonstrated to attenuate senescence-related cognitive deficits in SAMP8 mice by counteracting amyloid ß accumulation in the brain [302,303]. Flavones and flavonones are also significant components of bergamot juice and they confer anti-aging properties through the upregulation of SIRT1, Nrf2 and FOXO3 (that are involved in homeostasis, resistance to oxidative damage and overall health respectively), as it was demonstrated in models of senescent myocardiocytes and in vivo in mice [304]. 

Apigenin, also known as 5,7,4′-trihydroxyflavone, is a flavone typically found in parsley, oranges, and chamomile. Its ability to act as a metal chelator, free radical scavenger, and regulator of the main pathways involved in redox homeostasis [i.e., Nrf2, NF-kB, MAPK (mitogen-activated protein kinase) and Akt (a.k.a. protein kinase B] has increased its interest as an antioxidant molecule [305]. For example, creams rich in apigenin are used for their beneficial effects on skin aging prevention [306,307]. In vitro, human embryonic lung fibroblasts exposed to the pro-senescence stimuli H_2_O_2_ or doxorubicin, and subsequently treated with apigenin, show reduced SA-β gal activity, cell cycle promotion, increased levels of SIRT1, CAT and SOD and reduced expression of the senescence associated p21, p53 and p16 proteins compared to the untreated counterpart [308]. Similar results have been obtained in vivo following administration of apigenin daily for 8 weeks to a d-galactose-induced aging mouse model [309]. Moreover, thanks to its ability to inhibit the SASP and to interfere with the anti-apoptotic pathways, which are generally upregulated in senescent cancer cells, apigenin has been proposed as an adjuvant therapy for tumours, with promising results [237,310,311]. 

**Anthocyanins**. Bilberry and mulberry are considered promising nutrients for healthy aging because of their antioxidant properties related to anthocyanins that consist, among others, in the increase of SOD activity and AMPK (AMP-activated protein kinase)-mTOR autophagy pathway [312]. It has been reported that anthocyanins contrast senescence as they promote neural stem cells proliferation and diminish aging-related markers and cognitive impairment in mice [313]. The ability of anthocyanins to inhibit β amyloid aggregation is also of interest in therapeutic approaches aimed at slowing down cognitive decline [314]. In rats, the effect of mulberry extract was observed on the cardiovascular system, as it reduced the signs of senescence in endothelial cells [315,316]. 

Overall, a diet rich in these natural antioxidants may have a significant anti-aging effect. An indirect confirmation of this concept could be deduced by the fact that the Mediterranean Diet, which widely includes both flavanols, flavanones, flavons and anthocyanins, is characterized by well-known beneficial effects on health, including a healthy aging as it hinders the pathogenesis of many chronic diseases and extends life expectancy [317,318].

### 2.8. Non-Flavonoids

Non-flavonoid antioxidant substances, namely stilbenes (resveratrol), phenolic acids, curcuminoids (curcumin), and lignans [319] could be employed as anti-aging agents, acting against OS, inflammation, and cellular senescence (Table 4) [320,321,322,323,324,325,326,327,328,329,330,331,332]. 

**Stilbenes** are a family of natural phenolic compounds found in many plant species capable of acting as antioxidants, anti-inflammatory, antibacterial, and anticancer agents [333,334]. The most important and well-known stilbene is resveratrol (RSV), a phytoalexin found in black grapes, peanuts, blackberries, red wine, and various herbal remedies [335,336,337]. It has many biological properties, including antioxidant, and anti-inflammatory effects [320,338]. As an antioxidant agent, RSV can scavenge free radicals and reduce ROS formation, by inhibiting the expression of NADPH and glycogen synthase kinase 3 beta proteins, and upregulating the expression of some antioxidant enzymes, such as SOD2, CAT, GPx, and thioredoxin [320,339,340]. It can also stimulate the production of HO-1 by activating Nrf2 [341]. Resveratrol treatment has also been shown to prevent or slow down the progression of cardiovascular, neurological, and metabolic disorders, as well as to be promising in the prevention of cancer, viral infection, and pathological inflammation [342]. The activation of the anti-aging protein SIRT1 by RSV is thought to be responsible for its antioxidant and anti-inflammatory properties, as well as for some of its protective effects [343,344,345]. Interestingly, this compound possesses anti-aging properties, modulating OS, inflammation, and cellular senescence [346]. It has been demonstrated that RSV can reduce oxidative damage in the brain of aged mice by increasing the levels of SOD and plasma GPx, decreasing malondialdehyde, and lowering the expression of several pro-inflammatory proteins (IL1β and tumour necrosis factor α) in old mice, as well as in patients with coronary artery disease [321,322]. Overall, these studies suggest that RSV can be a tool useful in preventing diseases and damages associated with aging. Furthermore, it can also be a valid strategy for counteracting bone fragility and skin aging [347,348]. 

Although RSV’s antioxidant properties have been widely demonstrated, some studies [349,350] have highlighted its ability to also act as a pro-oxidant molecule. This dual role depends upon cell type, used dosage, and exposure time [336]. Interestingly, RSV, which acts as a pro-oxidant agent at high doses, can be a cancer chemopreventive agent by promoting tumour cell senescence [351].

**Phenolic acids** are organic compounds commonly found in a variety of plant-based foods and beverages. They have numerous health properties (anti-inflammatory, anticarcinogenic, antibacterial), and their ability to act as antioxidants makes them an effective weapon against chronic diseases [352]. They are divided into two classes: hydroxybenzoic (including gallic and ellagic acids) and hydroxycinnamic acids (including ferulic and p-coumaric acids) [319]. 

Gallic acid (GA) is a natural substance found in berries, gallnuts, grapes, fruits, and wine [353]. Many studies have suggested the beneficial properties of this molecule [354,355,356,357]. Furthermore, thanks to its antioxidant activity, GA has numerous applications, especially in cosmetic and medical areas where it can be used as a UVB protective agent [358], by decreasing the production of MMP-1 and IL-6 and increasing the expression of elastin, type I procollagen and transforming growth factor β1 [324], and as a nutritional supplement to protect cells from oxidative damage [359]. Interestingly, in addition to these positive qualities, GA could be a protective anti-aging agent, able to counteract cellular senescence. Indeed, it has been shown that GA can reduce senescence markers in rat embryonic fibroblast cells, delay thymus involution in old mice, and protect cardiac cells from oxidative damage and senescence, enhancing GST expression [296,323,353]. Furthermore, as mentioned above, this acid is widely employed as a component of skincare products in the cosmetic branch. For example, the synergistic action of gallic, ellagic, and chebulinic acids confers to some cosmetic constituents, such as triphala (an ayurvedic herbal rasayana formula), antioxidant, anti-inflammatory, and anti-aging properties on human skin cells, increasing the mRNA expression of collagen-I, elastin, filaggrin, involucrin, as well as SOD2 and aquaporin-3, and decreasing the levels of tyrosinase [360]. 

Ellagic acid (EA), in addition to acting in combination with other phenolic acids, can perform numerous functions on its own. It is found in a variety of fruits and vegetables, including strawberries, walnuts, and grapes, and it has important antioxidant, anti-inflammatory, antiviral and anticarcinogenic properties [361,362]. Its beneficial antioxidant activity has been reported in numerous studies [363,364]. As an antioxidant agent, EA can activate cellular antioxidant enzymes, like SOD, CAT and GPx, protect DNA from ROS and chelate metal ions [365]. Additionally, EA could also act as an anti-aging agent [365]. Treatment with EA can reduce liver and brain damage in aged rats [325] and may display an anti-photoaging effect on the skin by restoring SOD and total GSH activity and increasing Nrf2 expression [361]. Interestingly, the consumption of walnuts, which contain EA and other neuroprotective compounds, has been shown to improve memory impairment and protect against AD [366]. 

Ferulic acid (FA) is an anti-inflammatory [367], anti-cancer [368], antithrombotic [369], antibacterial [370], and radioprotective agent [371] found in fruits (grapes), vegetables (spinach, rhubarb, carrots, eggplants), grain, and cereal seeds (rye, barley, and oats) [372]. Thanks to its antioxidants, anti-diabetic, and neuroprotective properties [373,374], it has been shown to prevent type 2 diabetes and AD [375,376] by regulating antioxidant enzymes and caspase activities [326]. Acting as an antioxidant, FA can inhibit the enzymes that lead to ROS formation, scavenge free radicals, and promote the antioxidant enzymes activity [372]. This makes FA a compound widely used in cosmetics and in food industry, especially as an anti-aging agent [377]. It has been demonstrated to protect skin from UV radiation through its capacity to reduce the activity of the stress-inducible protein Gadd45 α, the expression of MMP-1 and MMP-3 mRNAs, as well as enhancing the levels of the antioxidant enzymes SOD1 and CAT [326,378]. As a result of its anti-aging properties, it could be an excellent cosmetic component for face masks and antioxidant and protective creams. Moreover, it is used in skin-lightening lotions, inhibiting tyrosinase activity and melanocytic proliferation [372]. Peanuts also contain FA, which may partly explain their ability to prevent aging and cognitive decline [379].

p-coumaric acid (p-CA) is a dietary compound widely found in oranges, apples, grapes, kiwis, onions, potatoes, eggplant, beans, and grains [380,381], which is endowed with antibacterial [382], anti-diabetic [383], anti-cancer [384], and radioprotective properties [385]. Furthermore, p-CA has analgesic, antipyretic, and anxiolytic effects, as well as the ability to inhibit platelet aggregation [381]. Being an antioxidant agent, the treatment with this acid promotes the expression of Nrf2, with the consequent increased levels of some antioxidant enzymes, including HO-1, SOD, NAD(P)H quinone dehydrogenase 1, CAT, and GPx [328]. Coumaric acid can also slow down the aging processes, due to its antioxidant and anti-inflammatory effects. It has been shown that p-CA can have beneficial effects on skin aging by decreasing collagenase, elastase, and hyaluronidase activity [386], and by reducing the inflammatory response and chondrocytes senescence, inhibiting MAPK and NF-kB signalling pathways [327]. However, although some works have demonstrated the antioxidant property of p-CA, Pieńkowska et al. highlighted that this acid is unable to counteract the premature senescence of human fibroblasts [387]. Consequently, the presence of contradictory evidence in literature requires further research.

**Curcuminoids** are natural polyphenolic compounds used as spices and food additives thanks to their aromatic and colouring properties [388]. The numerous beneficial activities make them potential supportive therapeutics for cancer and inflammatory bowel diseases [389]. Curcumin (CUR), a yellow phenolic pigment, commonly used as a food spice and herbal remedy, is the most well-known curcuminoid [390]. This compound is known to have anti-cancer [391], anti-bacterial [392], anti-diabetic [393], and cytoprotective activities [394]. Further, CUR possesses antioxidant properties, through which it can prevent lipid peroxidation, stabilize Nrf2, with the consequent expression of HO-1, and increase the levels of antioxidant enzymes, such as SOD, GST, GSH and GPx [395]. It has been suggested that this natural compound possesses therapeutic features in several malfunctions, including neurological, cardiological, and metabolic disorders, as well as ulcers, arthritis, acne, and dyspepsia [396]. Curcumin is also thought to be a useful anti-aging agent [395]. Indeed, it has been shown to improve cognitive deficits, suppress vascular aging and inflammation in elderly mice, and attenuate neuronal aging both in vitro and in vivo by downregulating the expression of p16 and p21 and upregulating antioxidant enzymes, including SOD1, CAT and GPx [329,330,397]. Furthermore, CUR supplementation has positive benefits on age-related disorders [398,399,400]. Although an antioxidant action in aging has been widely demonstrated, conflicting studies are present in literature regarding its inability to counteract OS [401], therefore more research is needed to better understand its antioxidant role. 

**Lignans** are found in many plant families and foods, including fruits, vegetables, nuts (sesame), grains, and seeds [402]. In addition to their numerous biological activities (antioxidant, anti-inflammatory, and antitumoral), as well as their ability to protect against the onset of chronic and metabolic diseases, lignans and their derivatives are also known to act as anti-aging agents [403]. In fact, they can suppress aging phenotypes in Drosophila adults, inhibit NADPH oxidase activity and upregulate antioxidant genes, such as SOD1, SOD2, catalase, and DNA repairing genes [221,404]. Further, they can reduce the levels of senescence in old human diploid fibroblasts, activating AMPK pathway [332]. Moreover, lignans molecules can protect tissues and organs against OS, inflammation, and senescence by acting as neuroprotective and radioprotective agents [405,406].

### 2.9. Minerals

Despite their tiny amount, micronutrients, i.e., vitamins and minerals, are essential for human health, exerting numerous functions, including antioxidant defence ranging from genome-related processes, such as DNA replication and repair, to metabolic processes and antioxidant defence [407,408]. Concerning the latter, the structural and functional roles of a few minerals such as zinc (Zn), selenium (Se), magnesium (Mg) copper (Cu) and manganese (Mn) is crucial (Table 5) [409,410,411,412,413,414,415,416,417,418,419,420].

**Zinc**, the second most abundant trace mineral in the body after iron, is involved in a wide range of key biological functions exerted through its catalytic role in enzymes, structural function in proteins and other cellular components [421,422,423]. Importantly, Zn exerts antioxidant functions through its catalytic action in Zn- SOD, via the formation and stabilization of sulfhydryl groups in proteins, thus maintaining membrane integrity and protecting it from oxidation, and through regulation of Zn-binding protein metallothionein expression. In this respect, evidence showed that Zn deficiency causes destabilization of membrane structure and augments OS [424,425,426,427,428]. In addition, Zn suppresses anti-inflammatory responses that would otherwise promote OS [425]. In vitro studies have shown that Zn deficiency is associated with an increased production of ROS, oxidative damage to DNA, proteins and lipids, destabilization of membrane structure, dysregulation of Zn-binding protein metallothionein [426,429]. For instance, in a colon cancer cell line [409] and dermal fibroblasts [410], Zn dysregulation promoted cellular senescence activating stress response and pro-apoptosis pathways. Some Zn-finger proteins and Zn-dependent enzymes, such as PATZ1 [430], ZKSCAN3 [431], ZHX3 [432], KLF4 [433] or Zfp637 [434] might be responsible for this Zn-mediated cellular senescence inhibition, reducing ROS production, DNA damage and telomere shortening (Table 6) [430,431,432,433,434,435,436,437]. In support of this concept, there is evidence that the downregulation of the Zn-finger protein ZEB2 significantly promotes cell senescence in hepatic stellate cells and dermal fibroblasts, limiting the development of fibrosis [435,436]. Again, the Zn-finger protein 768 has been found to be overexpressed in cancer cells, contributing to cell proliferation and repressing senescence [437]. On the other hand, Zn overload can contribute to augment cellular OS and senescence through different mechanisms not yet well-defined, but possibly related to organelles dysfunction [411,427,438].

**Selenium** relevance in human body is primarily due to its structural and catalytic roles in selenoproteins involved in redox signalling and homeostasis. Most of the human selenoproteins are oxidoreductases containing the amino acid selenocysteine (SeCys) at their catalytic site [439]. In the antioxidant enzymes GPXs, SeCys residues catalyse the reduction of hydrogen peroxide and peroxide radicals using glutathione as a substrate, thus lowering free radicals and consequent DNA damage [440,441]. A second crucial family of proteins containing SeCys and involved in redox biology is thioredoxin reductases (TRs), which contribute to the regulation of gene expression of multiple transcription factors implicated in inflammatory and cell cycle pathways (e.g., NF-kB and p53) [442], as well as in the recycling of antioxidant molecules [441].

Several in vitro studies have shown that Se supplementation counteracts senescence processes. For instance, in bone marrow stromal cells [443], cultured human fibroblasts [412], and keratinocytes [413], Se supplementation reduces ROS levels, DNA damage, telomere shortening and senescence biomarkers. In support of this concept, cells deficient in Selenoprotein H (a nuclear protein) displayed high levels of OS, persistent DNA damage and a decreased content in antioxidant molecules (glutathione) [414]. Furthermore, Se deprivation in mice has been found to accelerate DNA damage, senescence, and aging-related processes [415]. 

**Magnesium** has structural roles in DNA, proteins and enzymes including telomerases. It promotes DNA replication and transcription, protein synthesis and mitochondrial functions [444,445]. Low Mg can favour cellular senescence, accelerate telomerase shortening and disturb DNA stability, protein synthesis and mitochondrial function. Cell culture studies have demonstrated that Mg shortage negatively impact antioxidant defence, cell cycle progression and cellular viability; in particular, its deficiency in endothelial cell cultures enhances free radicals production and cell apoptosis [416], and increases the release of pro-inflammatory molecules [417]. Further, an enhanced production of hydrogenase peroxide and oxidative damage were measured in Mg-deficient embryo-hepatocytes [418]. Additionally, human fibroblasts cultured in Mg-deficient conditions showed a rapid telomere shortening and a decreased replicative lifespan [419]. Accordingly, animal studies have confirmed that a long-term Mg-deficient diet disrupts the redox balance and homeostasis and increases inflammation, consequently exacerbating the development and progression of aging-related diseases such as cardiovascular diseases, hypertension, or diabetes [420,446].

**Manganese** and **copper** are further trace elements involved in antioxidant mechanisms through their structural role within SOD and other crucial enzymes useful for protecting cells from OS [447,448,449]. Despite some controversial data arise, other findings point out that Mn supplementation might improve antioxidant functions in the lungs and ameliorate asthma conditions, as reported in a recent review [449].

Taken together, evidence from human studies is still lacking, but it is undeniable that proper mineral levels are crucial for the maintenance of the redox balance. However, it should be emphasized that a narrow range exists between the therapeutic and the pro-oxidative effects of some metals, including Se, Mn, Mg, Cu and Zn; therefore, a cautious and rational supplement choice must be assessed by the experts in the field. 

### 2.10. Others—Melatonin

Melatonin is a hormone produced and released by the pineal gland with immunomodulatory, oncostatic, anti-aging, and endocrine modulator functions [450,451]. Its antioxidant and anti-inflammatory activities are exerted through the suppression of cyclooxygenase 2, NLRP3 inflammasome, gasdermin D, TLR-4, NF-kB, and NO release, as well as the concomitant activation of SIRT1 and Nrf2 free radical scavenging network [452]. For example, inhibition of sodium nitroprusside-mediated NO release and increased production of transcripts coding for antioxidant enzymes (i.e., SOD1, GPx1 and CAT) have been reported upon melatonin treatment in neuroblastoma cells and HUVEC, respectively [451,453]. However, melatonin can also stimulate the release of pro-inflammatory factors depending on the concurrent conditions, although this response seems to be limited to early treatment stages and needs to be better investigated [454]. 

As a reduction in melatonin secretion has been observed during aging [452], several studies have explored the role of this hormone in counteracting cellular senescence [451]. In this respect, melatonin has been proven capable of reducing oxidative stress and replicative senescence by enhancing autophagy, activating AMPK/FOXO3 pathways and increasing mitochondrial membrane potential, both in vitro and in vivo [455,456,457]. Melatonin-induced decrease in p53, p21 and p16 proteins, together with enhanced SIRT1 activity, have also been reported in the context of H_2_O_2_-induced senescence [458,459]. Regarding MSCs, it is known that long term culture stimulates ROS generation, thus promoting oxidative stress-induced senescence [460]. In this context, decreased cellular senescence, preserved self-renewal and activation of antioxidant defence pathways have all been observed after melatonin supplementation [460,461]. In vivo, melatonin intake has been reported to diminish age-derived inflammation and apoptosis and to decrease lipid peroxidation, thiobarbituric acid reactive substances and protein carbonyls, thus counteracting hippocampal senescence and exerting an anti-aging and antioxidant effect on mice brains [462,463,464,465]. However, contrasting evidence remains about the effect of melatonin on the regulation of antioxidant enzymes [457,463,464,465]. Finally, brain oxidative stress amelioration and increased osteopontin and senescence marker protein-30 have been shown following melatonin treatment in the context of vascular demented rats [466]. Altogether, these findings suggest that, despite encouraging evidence, more research is needed to address the role of melatonin in vivo and identify possible side effects.

## 3. Discussion

The elderly population is growing exponentially in parallel with basic and clinical research discoveries and improvement of the quality of care for those who are sick. Aging is one of the risk factors for chronic diseases, atherosclerosis, cardiovascular diseases, stroke, kidney failure, chronic lung disease, cancers, diabetes, osteoporosis, arthritis, blindness, dementia, and neurodegenerative diseases [467,468]. Lifespans have increased dramatically over the last century in large part due to advances in medicine that have nearly eliminated certain deadly infectious diseases. 

Nutrition is one of the factors that can influence aging. Interviews of older persons reveal that most of them have continuous physical activities, positive thinking, and eat healthy foods such as vegetables, fruits, fishes, and less meat. It was shown that less calories [469], good sleep [470], less stress [471], good relationships [472], no smoke and low alcohol drinking are environmental factors capable of delaying aging, while genetic factors play a role for in 25% to 30% of life expectancy [473]. In this context, finding anti-aging drugs that meet the safety and effectiveness of long-term use has always been an important strategy for intervention in the aging field. 

Although cellular senescence is crucial for the proper functioning of several physiological processes, much scientific evidence has demonstrated that it also plays a leading role in the pathophysiology of aging and age-related diseases [3,474]. Because OS is an important senescence-triggering stimulus [13], the thorough investigation of existing antioxidants and the search for new ones are of major interest. In this narrative review, we have summarized the potential of the major classes of antioxidants in extending life and preventing senescence. However, most antioxidants are known to exert a dual effect (both pro-oxidant and antioxidant), especially based on the doses administered. Accordingly, it has been reported that high doses of antioxidants promote cellular senescence [156,475,476]. In this respect, more research is needed to define the optimal dosage of antioxidants, also considering the interaction of multiple compounds coming either from the diet or from supplements. Currently this remains a limit, as most of the studies investigated a single compound on a particular cell line or on a specific animal model, which often prevents generalizing the results in a broader context. Besides the dosage, a careful evaluation of the optimal administration window can be also crucial to achieve a clinical benefit as antioxidants efficacy might depend upon OS levels [81]. From a human perspective, not only dose assessment but also the time of initiation (childhood, adolescence, adulthood, old age) and the duration of the treatment (lifelong, at alternating intervals or for a defined period) might affect the clinical outcomes. There are still few studies on the synergy and interference of multiple antioxidants taken in combination—increasingly fashionable in the modern society—and the benefits and risks of this approach should be carefully evaluated. Similarly, when integrated as supplements, antioxidants may give rise to different effects depending upon the time of the day they are taken and individual differences such as physical activity and lifestyle, which may also greatly vary among subjects. Given these considerations, studies conducted in vitro under limited variable conditions should be reproduced and validated on large-cohorts clinical trials. 

Current reports distinguish the existence of senolytic (able to remove senescent cells) or senomorphic (able to modulate senescent cells) substances, which can include some plant-derived antioxidants (e.g., quercein, fisetin) but usually are functional concepts, the description of which is outside our review remit, and which should encourage clinical research and nutraceutical application, more than in vitro investigations [266,477,478,479]. It is difficult, to date, to indicate if an anti-oxidant vitamin is senolytic or senomorphic, due to the huge complexity of biological phenomena.

## 4. Conclusions

Antioxidants are formidable substances, mostly derived from plants, which have been considered so far to be beneficial agents able to address many redox-mediated injuries. Aging is usually considered as a major playground of oxidative stress, but it should be highlighted that ROS are fundamentally signalling molecules and that most of the stress responding mechanisms are tuned by fine modulation of ROS as signalling agents. Therefore, a correct action to address senescence is to find approaches and methods to improve and promote this modulation. Wise people used to state that equilibria stand on the fine regulation of pro- and con- hallmarks of xenobiotics. This is also our wish and recommendation for the future. 

## Figures and Tables

**Table 1:** CoenzymeQ_10_ and ubiquinol in the prevention of OS-induced senescence.

Ref.	Study Design	Treatment	Form	Results	Conclusion
Chen et al., 2019 [59]	HAEC chronically treated with NRTI	5 μM CoQ_10_ continuously applied across passages	CoQ_10_	↓ NRTI-induced senescence ↓ ROS ↑ Mitochondrial respiration rate	CoQ_10_ reduces cardiovascular side effects of NRTI treatment
Tarry-Adkins et al., 2013 [58]	Mouse model of low birth-weight and catch-up growth	Post-weaning dietary supplement	CoQ_10_	↓ Nitrosative and OS ↓ DNA damage ↓ Cellular senescence ↓Telomere shortening ↓ Apoptosis	CoQ_10_ prevents cardiac aging and cardiovascular risk
Ma et al., 2014 [60]	PS-1-mutated AD fibroblasts	Medium with 50 μg/mL WS-CoQ_10_	WS-CoQ_10_	↓ ROS ↑ Cell doubling ↓ SIPS ↑ PCNA expression ↓ MnSOD, p21, p16Ink4A, and Rb ↑ Autophagy	WS-CoQ_10_ inhibits SIPS and improves autophagy
Xue et al., 2017 [61]	Mouse PSCs	Cell treatment with 1/10/100 μM CoQ_10_ for 24/48/72 h	CoQ_10_	↓ Apoptosis ↓ SA-*β*-Gal ↓ ROS and malondialdehyde after 72 h treatment	CoQ_10_ may act as a target in PSC-related pathologies
Wu et al., 2020 [62]	ORX mice	CoQ_10_ 50 mg/kg/day	CoQ_10_	↓ OS ↓ Cell senescence ↑ Osteoblastic bone formation	CoQ_10_ is anti-osteoporosis and -senescence
Mine et al., 2021 [63]	H_2_O_2_-induced SIPS in human skin fibroblasts	1 μM and 10 μM CoQ_10_	CoQ_10_	↑ Cell viability ↓ OS ↓ SA-*β*-Gal ↓ SASP	CoQ_10_ can contribute to increase lifespan
Zhang et al., 2015 [64]	D-galactose -induced aging in MSC	1/10/100 mmol/L CoQ_10_ for 48 h	CoQ_10_	↓ p-AKT and p-mTOR ↓ MSC senescence	CoQ_10_ inhibits MSC senescence and aging
Velichokovska et al., 2019 [65]	NPCs exposed to ART	NP-based delivery of CoQ_10_ to mitochondria	CoQ_10_	↓ ROS ↑ SIRT3 ↑ Cell proliferation ↓ SA-*β*-Gal ↑ Telomere length	ART-induced senescence can be reversed by NP-CoQ_10_
Marcheggiani et al., 2021 [66]	CoQ_10_-deprived HDF	5, 10 or 15 μg/mL of either CoQ_10_ or CoQ_10_H_2_ for 24 h	CoQ_10_ or CoQ_10_H_2_	↓ SA-*β*-Gal ↓ OS ↓ p21 ↑ Elastin, collagen type I	CoQ_10_ or CoQ_10_H_2_ prevent skin aging and support skin vitality
Huo et al., 2018 [67]	HUVEC treated with H_2_O_2_	24 h in medium with 10 *μ*M CoQ_10_H_2_	CoQ_10_H_2_	↓ SA-*β*-Gal positive cells ↓ SASP ↓ ROS ↓ Apoptosis ↑ NO and eNOS ↑ Mitochondrial function	CoQ_10_H_2_ delays vascular aging
Yan et al., 2006 [68]	SAMP1 mice	250 mg/kg/day lifelong supplement	CoQ_10_H_2_	↓ Senescence grading scores ↑ Female body weight = Lifespan = Urinary 8-OHdG and acrolein-lysine adduct	CoQ_10_H_2_ decreases cellular senescence in middle-aged SAMP1 mice
Olivieri et al., 2013 [69]	Senescent HUVECs in presence or absence of LPS	10 µM CoQ_10_H_2_ for 24 h or 60 days	CoQ_10_H_2_	↓ LPS-induced NF-kB activation ↓ SASP	CoQ_10_H_2_ may prevent aged-induced endothelial dysfunction
Maruoka et al., 2014 [70]	SAMP1 mice	300 mg/kg (Group A) or 30 mg/kg CoQ_10_H_2_ (Group B)	CoQ_10_H_2_	↓ Senescence score at 10 months in Group A compared to B ↓ OS ↑ Antioxidant potential	CoQ_10_H_2_ reduces senescence and OS in a dose-dependent manner
Schmelzer et al., 2010 [71]	Middle aged SAMP1 mice	500 mg/kg/day of CoQ_10_H_2_ for 6 or 14 months	CoQ_10_H_2_	↓ Senescence grading score ↑ PPAR-alpha	CoQ_10_H_2_ decelerates degeneration in SAMP1 mice
Cirilli et al., 2020 [72]	HUVEC treated with CSE for 24 h	10 µM CoQ_10_H_2_	CoQ_10_H_2_ and menaquinone 7	↓ OS ↓ Inflammation ↓ Apoptosis ↓ SA-*β*-Gal	CoQ_10_H_2_ and menaquinone-7 counteract CSE-induced damage
Tian et al., 2014 [73]	SAMP1 mice	Dietary CoQ_10_H_2_ (0.3% *w*/*w*)	CoQ_10_H_2_	↑ PGC-1α, SOD2, IDH2, SIRT1, SIRT3 ↑ Mitochondrial complex I activity ↓ OS ↑ GSH/GSSG ratio	CoQ_10_H_2_ protects against aging progression

Abbreviations: AD: Alzheimer’s Disease; ART: antiretroviral therapy; CoQ_10_: coenzyme Q_10_; CoQ_10_H_2_: ubiquinol; CSE: cigarette smoke extract; eNOS: endothelial nitric oxide synthase; GSH: reduced glutathione; GSSG: oxidized glutathione; HAEC: human aortic endothelial cells; HDF: human dermal fibroblasts; HUVEC: human umbilical vein endothelial cells; IDH2: isocitrate dehydrogenase 2; LPS: lipopolysaccharide; MSC: mesenchymal stem cell; NF-kB: nuclear factor kappa-light-chain-enhancer of activated B cells; NO: nitric oxide; NP: nanoparticle; NPCs: neural progenitor cells; NRTI: nucleoside reverse transcriptase inhibitors; ORX: orchiectomized; OS: oxidative stress; PCNA: proliferating cell nuclear antigen; PGC-1α: peroxisome proliferator-activated receptor γ coactivator 1α; PPAR: peroxisome proliferator-activated receptor; PS-1: presenilin-1; PSCs: pancreatic stellate cells; ROS: reactive oxygen species; SA-*β*-Gal: senescence-associated *β*-galactosidase; SAMP1: one of the senescence accelerated mice (SAM) strains, which shows shortened life span and early signs of senescence; SASP: senescence-associated secretory phenotype; SIPS: stress induced premature senescence; SIRT: sirtuin; SOD2: superoxide dismutase 2; WS-CoQ_10_: water-soluble CoQ_10_; 8-OHdG: 8-hydroxy-2′-deoxyguanosine; ↑: increase; ↓: decrease.

**Table 2:** SkQ1 and MB in the prevention of OS-induced senescence.

Compound	Sample	Treatment	Results	Ref.
SkQ1	*Podospora anserina*, *Ceriodaphnia affinis*, *Drosophila melanogaster*, and mouse	Nano- and subnanomolar concentrations of SkQ1	-Prolonged lifespan -Reduced senescence	[74,75]
SkQ1	Wistar and senescence-accelerated rats	250 nmol per kg/day SkQ1 (starting from 19 months of age)	-Reduced and reversed age-related decline	[76]
SkQ1	BALB/c and C57BL/6 mice	Lifelong administration of SkQ1	-Decreased cardiomyopathy, fibrosis and cardiac hypertrophy	[77]
SkQ1	Senescence-accelerated rats	250 nmol/kg body weight, daily (from 1.5 to 23 months of age)	-Reduced Alzheimer’s disease pathology	[78]
MB	Human IMR90 fibroblasts	10, 100 or 1000 nM of MB for 4 days	-Delayed senescence -Improved mitochondrial function	[79]
MB	Human skin fibroblasts derived from progeria patients	100 nM MB	-Effective ROS scavenging -Improved skin fibroblast proliferation -Delayed senescence	[80]
MB	Human bone marrow-derived MSCs	200 nM MB	-Improved expansion in vigorous MSCs -Improved differentiation in vigorous MSCs	[81]
MB	Primary rat RGCs	1 μM and 10 μM MB	-Stimulated mitochondrial function -Enhanced neuroprotection	[82]

Abbreviations: MB: methylene blue; MSCs: mesenchymal stem cells; RGCs: retinal ganglion cells.

**Table 3:** Preclinical studies on flavonoids in aging.

Type of Flavonoid	Effect	Reference
4,4′-dimethoxychalcone	- Increases lifespan (yeast, worms, and flies) - Reduces human cell senescence	- [229]
Naringenin	- Antioxidant effects - Reduces cardiovascular damage - Prolongs lifespan in flies	- [230]
Nobiletin: Rutaceae family	- Antioxidant effects	- [231]
Quercetin	- Blocks senescence of endothelial cells - Reduces expression of senescence-associated secretion phenotype (SASP) - Enhances health span and lifespan in old mice. - Improves cardiovascular diseases - In combination with dasatinib improves 6-min walking distance, speed, and ability to stand up	- [232] - [233] - [234] - [228]
Fisetin	- Blocks cultured senescent fibroblasts in human and animal - Increases lifespan	- [235] - [236]
Apigenin	- Reduces SASP	- [237]
Theaflavin	- Decreases cell senescence - Increases lifespan	- [238] - [239]
Myrecitin	- Increases mitochondria metabolism - Reduces neurotoxicity	- [240] - [241]
Rutin	- Reduces oxidative stress - Reduces cell senescence - Reduces production of proinflammatory cytokines - Reduces metabolic disorders	- [242] - [243] - [244]
Luteonil	- Reduces human senescence cells - Reduces expression of SASP	- [245]
Kaempferol	- Reduces SASP - Reduces oxidative stress	- [229]
Hesperidin	- Reduces oxidative stress - Increases antioxidant enzymes	- [246] - [247]
Dyhydromericetin	- Reduces oxidative stress - Reduces inflammation - Increases cognitive function - Reduces gut dysfunction	- [248] - [249] - [250]
Epicatechin	- Reduces cell senescence - Increases brain function - Reduces skeletal muscle dysfunction	- [251] - [252] - [253]
Genistein	- Decreases pro-inflammatory genes expression - Decreases cell senescence - Increases brain cognitive function	- [254] - [255] - [256]

**Table 4:** Effects of non-flavonoids treatment in different experimental studies.

Non-Flavonoid	Model	Effects	Reference
**Resveratrol**	HUVEC cells	- Prevention of cells apoptosis, by reducing oxidative damage (↑SOD, ↓ROS, and ↓MDA) and inhibiting mitochondrial pathway	[320]
	Senescence-accelerated mice	- Reduction of oxidative damage in the brain (↑SOD, ↑GSH-Px, and ↓MDA)	[321]
	Old male mice	- Decreasing of inflammation in the liver (↓IL-1β, ↓TNFα, and ↓COX2)	[322]
**Gallic acid**	Rat embryonic fibroblast cells	- Reduction of inflammation (↓NF-kB, ↓TNFα, ↓IL-1β, and ↓IL-6) - Reduction of beta-galactosidase activity - Decreasing of ROS production and lipid peroxidation	[323]
	UVB-irradiated human fibroblast cells	- Inhibition of MMP-1 and IL-6 expression, and increasing of procollagen type I	[324]
	UVB-irradiated hairless mice	- Prevention of wrinkle formation, by upregulating procollagen type I and elastin levels	
**Ellagic acid**	D-galactose-treated rats	- Attenuation of OS in liver and brain (↑SOD, ↑CAT, ↑GSH-Px) - Amelioration of histopathological changes - Inhibition of inflammation (↓IL-6, ↓IL-1β, ↓TNFα)	[325]
**Ferulic acid**	UVA-irradiated nHDF	- Increasing of proliferation and cell cycle - Reduction of OS (↑SOD1, and ↑CAT) - Inhibition of cellular senescence (↓p16)	[326]
**p-coumaric acid**	Rat chondrocytes	- Amelioration of inflammation and cellular senescence, by inhibiting MAPK and NF-kB pathways	[327]
	Mice fed with high-fat diet (HFD)	- Inhibition of ROS production, lipid peroxidation and upregulation of antioxidants enzymes (↑SOD, ↑CAT, ↑GSH-Px, ↑HO-1)	[328]
**Curcumin**	Senescence-accelerated mice	- Improvement of cognitive deficits, by decreasing OS (↑SOD) and increasing p-CaMKII and p-NMDAR1 expression	[329]
	Mice fed with HFD	- Decreasing of OS (↑HO-1) - Reduction of inflammation and vascular aging, by lowering the accumulation of senescent cells in the aorta and MCP-1 levels in the blood	[330]
**Lignans**	nPC12 cells	- Reduction of lipid peroxidation (↓COX-2), and ROS production	[331]
	D-galactose aging mice	- Attenuation of OS (↑SOD, ↑GPx)	
	Old HDFs	- Decreasing of senescence markers expression (cyclin D1, p16, p27, p21, caveolin-1), by activating AMPK pathway, and ROS levels	[332]

Abbreviations: AMPK: (AMP-activated protein kinase); CAT: (catalase); GPx: (glutathione peroxidase); GSH-Px: (plasma glutathione peroxidase); HO-1: (heme oxygenase 1); HUVEC: (Human umbilical vein endothelial cell); IL-1β: (interleukin 1 β); IL-6: (interleukin 6); MAPK: (mitogen-activated protein kinase); MCP-1: (monocyte chemoattractant protein-1); MDA: (malondialdehyde), COX-2 (cyclooxygenase 2); MMP-1: (matrix metalloproteinase 1); NF-kB: (nuclear factor kappa B); nHDF: (normal human dermal fibroblasts); nPC12: (neuronally differentiated phenchromocytoma cells); p-CaMKII: (p-calcium/calmodulin-dependent kinase II); p-NMDARI: (p-N-methyl-D-aspartate receptor subunit 1); p16: (cyclin-dependent kinase inhibitor 2A); p21: (cyclin-dependent kinase inhibitor 1); p27: (cyclin-dependent kinase inhibitor 1B); SOD: (superoxide dismutase), ROS (reactive oxygen species); SOD1: (superoxide dismutase 1); TNFα: (tumour necrosis factor); ↑: increase; ↓: decrease.

**Table 5:** Minerals as modulators of OS-induced senescence.

Mineral	Sample	Treatment/Condition	Result	Ref.
Zinc	Colon cancer lines SW480 and SW620	↓ Zinc	↑ Oxidative stress, cellular proliferation, stress signalling morphological changes, cell death	[409]
Zinc	Dermal fibroblast	↑ Zinc	↑ Oxidative stress and DNA damage	[410]
Zinc	HCAECs	↑ Zinc	↑ Senescence	[411]
Selenium	Bone marrow stromal cells	↑ Selenium	↓ Senescence	[412]
Selenium	Keratinocytes	↑ Selenium	↓ Senescence	[413]
Selenium	Human fibroblasts	↑ Selenium	↓ Senescence	[414]
Selenium	Mice	↓ Selenium	↑ Senescence	[415]
Magnesium	Endothelial cells	↓ Magnesium	↑ Oxidative stress and cell death	[416]
Magnesium	Endothelial cells	↓ Magnesium	↑ Pro-inflammatory molecules	[417]
Magnesium	Embryo-hepatocytes	↓ Magnesium	↑ Oxidative stress	[418]
Magnesium	Human fibroblasts	↓ Magnesium	↑ Telomere shortening	[419]
Magnesium	Rats	↓ Magnesium	↑ Age-related diseases	[420]

Abbreviations: HCAECs: Human coronary artery endothelial cells; ↑: increase; ↓: decrease.

**Table 6:** Interplay between zinc-finger proteins and senescence.

Mineral	Sample	Zinc-Finger Proteins	Ref.
Zinc	Endothelial cells	PATZ1 is downregulated in senescence	[430]
Zinc	Mesenchymal stem cells	ZKSCAN3 upregulation contrast senescence	[431]
Zinc	Human diploid fibroblast	ZHX3 is downregulated in senescence	[432]
Zinc	Mouse embryonic fibroblasts	KLF4 reduces cellular senescence and DNA damage	[433]
Zinc	NIH3T3 and C2C12 cells	ZFP637 protects from oxidative stress	[434]
Zinc	Hepatic stellate cells	ZEB2 protects from oxidative stress and senescence	[435]
Zinc	Dermal fibroblasts	ZEB1 protects from oxidative stress and senescence	[436]
Zinc	Cell lines (A549, NCI-H441 and NCI-H460, 293T)	ZNF768 depletion induces senescence	[437]

Abbreviations: KLF4: Kruppel-like factor 4; PATZ1: POZ/BTB and AT-hook-containing *z*inc finger protein 1; ZEB1: zinc finger E-box-binding homeobox 2; ZEB2: zinc finger E-box-binding homeobox 2; ZFP637: zinc finger protein 637; ZHX3: zinc fingers and homeoboxes 3; ZKSCAN3: zinc finger with KRAB and SCAN domains 3; ZNF768: zinc finger protein 768.

## Data Availability

Data is contained within the article.
